# siRNA targeting ANGPTL3 stands in the spotlight for lipid-lowering therapy

**DOI:** 10.1016/j.omtn.2023.03.020

**Published:** 2023-04-19

**Authors:** Bo Hu, Yuanyu Huang

**Affiliations:** 1 School of Life Science, Beijing Institute of Technology, Beijing 100081, China; 2 Advanced Research Institute of Multidisciplinary Science, Beijing Institute of Technology, Beijing 100081, China; 3 School of Medical Technology, Beijing Institute of Technology, Beijing 100081, China; 4 Key Laboratory of Molecular Medicine and Biotherapy, Beijing Institute of Technology, Beijing 100081, China; 5 Key Laboratory of Medical Molecule Science and Pharmaceutics Engineering, Beijing Institute of Technology, Beijing 100081, China; 6 School of Materials and the Environment, Beijing Institute of Technology, Zhuhai 519085, China; 7 Rigerna Therapeutics, Suzhou, Jiangsu 215127, China

Dyslipidemias such as hypercholesterolemia and hypertriglyceridemia are recognized as major risk factors for cardiovascular disease (CVD).^1^ The existing medications can significantly decrease the level of blood cholesterol (CHO),^2^ but effective therapy for blood triglyceride (TG) reduction is still a significant clinical unmet need. In a recent study published in *Molecular Therapy – Nucleic Acids*, Wang et al.^3^ reported a small interfering RNA (siRNA) termed ANGsiR10 as a novel therapeutic, achieving long-lasting reduction of TG and non-high density lipoprotein cholesterol (non-HDL-c) in both high TG mice and hyperlipidemic monkeys. This work potentially provides more benefits to patients with hyperlipidemia or CVD.

Angiopoietin-like protein 3 (ANGPTL3) plays an important role in lipoprotein regulation: it is synthesized exclusively in liver and then secreted into blood circulation.^4^ It was observed that the loss of function of ANGPTL3 in human beings leads to increased activity of lipoprotein lipase (LPL) and endothelial lipase (EL)^5^^,^^6^ and reduced plasma TG,^7^ high-density lipoprotein cholesterol (HDL-c), and low-density lipoprotein cholesterol (LDL-c). In addition, heterozygous loss-of-function mutations in the human ANGPTL3 gene are negatively associated with CVD risk.^8^ These scenarios make ANGPTL3 a promising target for the treatment of hyperlipidemia and CVD. To date, one medication has been approved for marketing by the FDA (evinacumab, antibody), and two medications are undergoing phase 2 investigation (vupanorsen, antisense oligonucleotide [ASO], and ARO-ANG3, siRNA). These therapeutics successfully reduced the level of ANGPTL3 in plasma and led to lower circulating lipid levels in the patients (Table 1).

**Table 1:** Comparison of different types of ANGPTL3 medications

Reduction	Antibody^a^	ASO^a^	ANGsiR10^b^
TC	✓	✓	✓
HDL-c	✓	✓	✕
Non-HDL-c	✓	✓	✓
TG	✓	✓	✓
ApoA	N/A	N/A	✓
ApoB	N/A	✓	✓

ASO, antisense oligonucleotide; TC, total cholesterol; HDL-c, high-density lipoprotein cholesterol; TG, triglyceride; ApoA, apolipoprotein A; ApoB, apolipoprotein B; N/A, not available.

a Data were collected from healthy volunteers.

b Data were collected from hyperlipidemic monkeys.

siRNA therapeutics are developed based on a Nobel Prize-wining science; they can downregulate the expression of any interested gene in a sequence-specific way by mediating targeted mRNA degradation. Modification geometry and delivery system are the fundamental technologies to determine the performance of siRNA modalities.^9^ Chemical modification of siRNA can significantly improve the stability and reduce/erase the risk of immunogenicity and off-target effects (as well as off-target-induced toxicity),^10^ while the delivery system is the determinant for achieving efficient transportation to target cells. So far, five therapeutics have been successfully commercialized (Table 2): the first one (Onpattro) employed lipid nanoparticle (LNP) as the delivery carrier, while the other four introduced trivalent N-acetylgalactosamine (GalNAc) conjugation on the sense strand of siRNA.

**Table 2:** Marketed siRNA therapeutics and their core information

Name	Indication	Modification	Delivery	Target	Dose regimen
Onpattro	TTR-mediated amyloidosis	2′-OMe, 2′-F	LNP (DLin-MC3-DMA)	TTR	0.3 mg/kg, q3w, i.v.
Givlaari	acute hepatic porphyrias	ESC	GalNAc conjugate	ALAS1	2.5 mg/kg, qm, s.c.
Oxlumo	primary hyperoxaluria type 1	ESC	GalNAc conjugate	HAO1	3 mg/kg, q3m, s.c.
Leqvio	hypercholesterolemia	ESC	GalNAc conjugate	PCSK9	284 mg, q3m, s.c. for 2 doses, followed by q6m
Amvuttra	TTR-mediated amyloidosis	ESC	GalNAc conjugate	TTR	25 mg, q3m, s.c.

2′-OMe, 2′-methoxy; 2′-F, 2′- fluoro; ESC, enhanced stabilization chemistry; TTR, transthyretin; HAO1, hydroxyacid oxidase 1; ALAS1, delta-aminolevulinate synthase 1; PCSK9, proprotein convertase subtilisin/kexin type 9; q3w, treatment once every 3 weeks; qm, treatment once every month; q3m, treatment once every 3 months; q6m, treatment once every 6 months; i.v., intravenous; s.c., subcutaneous.

In the present study, the authors firstly designed 25 siRNA sequences targeting human ANGPTL3 mRNA. Notably, these sequences were generated under the consideration of avoiding affecting highly homologous genes such ANGPTL4 and ANGPTL8, which is favorable to erase potential off-target effects. Subsequently, all siRNAs were armed with chemically modified monomers at specific positions of both strands and conjugated with trivalent GalNAc at the 3′ end of the sense strand. After *in vitro* and *in vivo* evaluation, ANGsiR10 was finally selected as the lead compound. It completely matches with ANGPTL3 mRNA transcripts of human, monkey, and mouse.

The authors then determined the efficacy and dose regimen of ANGsiR10. The high TG mice received a single subcutaneous injection at doses of 1 and 3 mg/kg, respectively. Continuous lipid monitoring data revealed that ANGsiR10 displayed a dose-dependent pattern. The highest reduction of TG was 96.3%, and total CHO (mainly contributed by the non-HDL-c) was 75.3% in the animals that received the 3 mg/kg siRNA dose. Particularly, a high dose of ANGsiR10 induced a 14-week reduction in blood lipids. In addition, three dose regimens were designed and explored, which demonstrated that 3 mg/kg siRNA with one dose every 4 weeks achieved superior treatment outcomes.

To further evaluate the efficacy of ANGsiR10, a study in a hyperlipidemic monkey model with spontaneous metabolic syndrome was performed. Two parameters, TG and non-HDL-c, were significantly decreased, and the reductions were observed during the whole period of the study. Besides, other beneficial effects such as reduced plasma apolipoproteins and body weight were also observed. The ANGPTL3 mRNA expression in the liver, and the protein level in the serum, were also measured. The results showed that ANGsiR10 efficiently suppressed the expression of mRNA and protein in animals, which proved that all treatment outcomes were mediated by the reduction of ANGPTL3. Finally, histopathology and serum biochemistry analysis suggested that ANGsiR10 exhibited a satisfactory safety profile.

Undeniably, siRNA drugs employing chemical modifications and GalNAc conjugate are the preferred solution for treating diseases caused by abnormally high expression of genes in hepatocytes. Given the specific expression of ANGPTL3 in hepatocytes and its biological function, ANGsiR10 can serve as an important game player for lipid lowering, even for the management of CVD and type 2 diabetes. Efficacy, safety, and patient compliance are important criteria to determine the performance of medication, ANGsiR10 may be outstanding in all of the above aspects. It can reduce TG levels for 14 weeks, while antibody drugs can only last for 5 weeks.^5^ Meanwhile, the level of non-HDL-c and apolipoproteins were all reduced in animals that received ANGsiR10. In addition, ANGsiR10 displayed ideal safety profiles in animals. A subcutaneous and up to monthly administration scheme can provide better patient compliance than existing lipid-lowering medications. In addition, four marketed siRNA drugs also demonstrated the same advantages. Compared with Onpattro, Amvuttra reduced the dose frequency from once every 3 weeks to once every 3 months with a similar dose. Leqvio can be administered every 6 months. These treatment outcomes are barely achieved by small molecules and protein/antibody drugs. Therefore, it is believed that siRNA will become a blockbuster modality in the pharmaceutical industry, and GalNAc-conjugated siRNAs are promising treatments for liver-originated diseases. Moreover, after the establishment of extrahepatic delivery platforms, siRNA therapeutics will further enter a new era.
